# Bilateral Salpingo-Oophorectomy Is Superior to Salpingectomy Alone in Preventing Non-Tubal Tumor Development in a Mouse Model of High-Grade Serous Carcinoma

**DOI:** 10.3390/cancers17172759

**Published:** 2025-08-24

**Authors:** Yali Zhai, Eric R. Fearon, Kathleen R. Cho

**Affiliations:** 1 Department of Pathology, University of Michigan Medical School, Ann Arbor, MI 48109, USA; yaliz@med.umich.edu (Y.Z.); fearon@med.umich.edu (E.R.F.); 2 Department of Internal Medicine, University of Michigan Medical School, Ann Arbor, MI 48109, USA; 3 Department of Human Genetics, University of Michigan Medical School, Ann Arbor, MI 48109, USA; 4 Rogel Cancer Center, University of Michigan, Ann Arbor, MI 48109, USA

**Keywords:** endosalpingiosis, risk-reducing surgery, genetically engineered mouse model, high-grade serous carcinoma, primary peritoneal

## Abstract

Background/Objectives: High-grade serous carcinoma (HGSC) is the leading cause of ovarian cancer-related mortality. It usually arises from fallopian tube epithelium, with a smaller subset arising in non-tubal sites including the ovary or peritoneum. The origin of HGSCs without evidence of tubal involvement remains unclear. Moreover, in women with genetic predisposition to developing HGSC, the additional protection afforded by prophylactic removal of the ovaries in addition to the fallopian tubes has not yet been established. Methods: We used a well-characterized genetically engineered mouse model (GEMM) of oviductal HGSC based on conditional, somatic inactivation of the *Brca1*, *Trp53*, *Rb1*, and *Nf1* tumor suppressor genes (*BPRN* mice) to compare preventive effects for HGSC via bilateral salpingectomy versus bilateral salpingo-oophorectomy. We also explored the origins of non-tubal HGSCs in ectopic tubal-type epithelium (endosalpingiosis) present in the mouse ovaries and peritoneum. Results: While bilateral salpingectomy significantly reduced the incidence of HGSCs in the GEMM model, bilateral salpingo-oophorectomy completely prevented tumor development. We identified an example of HGSC with apparent origin in endosalpingiosis, implicating endosalpingiosis as a likely precursor for non-tubal HGSC. Conclusions: Our findings confirm the superiority of bilateral salpingo-oophorectomy over salpingectomy alone in reducing HGSC risk and affirm the rationale for surgical strategies to reduce HGSC risk in women carrying pathogenic variants of *BRCA1/2* and other genes associated with homologous recombination deficiency. Our findings also illustrate how work with GEMMs can advance new insights into HGSC pathogenesis.

## Simple Summary

The most common type of ovarian cancer, high-grade serous carcinoma (HGSC), arises more often from the fallopian tube than the ovary. In women genetically predisposed to developing HGSC, prophylactic removal of their fallopian tubes and ovaries is known to greatly reduce cancer risk, but it is unclear how much benefit removal of the ovaries provides beyond removal of the fallopian tubes alone. In this study, mice genetically engineered to develop HGSC in their oviducts (mouse fallopian tubes) were used to compare preventive effects of removing the oviducts alone (bilateral salpingectomy, BS) with removal of oviducts and ovaries (bilateral salpingo-oophorectomy, BSO). Both procedures significantly reduced HGSC incidence, but only BSO completely prevented tumor development. In mice that developed ovarian cancer after BS, tumors appeared to originate from tubal-type epithelium residing in the ovary (endosalpingiosis), suggesting endosalpingiosis as a potential cell of origin for HGSCs arising outside of the fallopian tubes.

## 1. Introduction

It is now widely accepted that high-grade serous carcinoma (HGSC), the most common and most lethal type of “ovarian” cancer, usually arises from fallopian tube epithelium (FTE). However, up to a third of HGSCs lack evidence of a tubal origin, even after microscopic examination of all resected tubal tissue [1]. There are uncertainties regarding the cells from which non-tubal HGSCs arise, but it has been proposed that ovarian and/or primary peritoneal HGSCs might develop either from occult tubal precursors, from ovarian surface epithelium or benign serous ovarian tumors, or from normal or neoplastic FTE that becomes denuded and subsequently implanted in non-tubal sites, including the ovary and peritoneal surfaces [2,3,4,5]. It has also been proposed that latent cells present in ectopic locations outside of the tubes retain the capacity for multi-lineage differentiation and can reconstitute glands lined by tubal-type epithelium (Mullerian metaplasia hypothesis) [6,7]. Tubal-type tissue outside of the fallopian tube is referred to as endosalpingiosis and is comparable to its better-known endometrial counterpart—endometriosis. Endosalpingiosis is characterized by single or small groups of simple glands that are sometimes cystically dilated. The glands are lined by the same cell types present in normal FTE, including ciliated, secretory and intercalated (peg) cells. Endosalpingiosis is most frequently found on the pelvic peritoneum covering the uterus, fallopian tubes, ovaries, and cul de sac, and in pelvic lymph nodes and the omentum [8]. Endosalpingiosis is usually an incidental finding identified intraoperatively or more often microscopically during histopathologic review of surgical specimens. Because endosalpingiosis has uncertain clinical significance, its presence is often not mentioned in diagnostic pathology reports and hence its prevalence is unclear. One prospective study identified endosalpingiosis in roughly a quarter of surgically resected omental specimens [8]. In the human ovary, endosalpingiosis manifests as benign glands with tubal-type differentiation in the ovarian cortex. A subset of ovarian “inclusion” cysts are lined by tubal-type epithelium and can be considered cystic endosalpingiosis.

We have developed genetically engineered mouse models (GEMMs) of HGSC in which the *Ovgp1* promoter is used in transgenic mice to drive expression of a Tamoxifen (TAM)-regulated Cre recombinase protein. The Cre recombinase can be used to inactivate concurrently the *Brca1*, *Trp53*, *Rb1*, and *Nf1* (*BPRN*) tumor suppressor genes (TSGs) in the oviductal epithelium (equivalent to human FTE). Mice with the *Ovgp1-CreER^T2^* transgene and homozygous for floxed (fl) alleles of *Brca1*, *Trp53*, *Rb1*, and *Nf1* are referred to here as “*BPRN*” mice. *BPRN* mice treated transiently with TAM dosing at 6–8 weeks of age develop oviductal tumors that closely mimic their human HGSC counterparts with respect to associated precursor lesions, tumor morphology, and various biological features [9,10]. The murine oviductal HGSCs mirror the genomic alterations, gene expression profiles, and immune microenvironment of human HGSCs, based on an integrated molecular analysis of the mouse tumors with comparison to human HGSCs [11]. In the *BPRN* GEMM model, the earliest detectable oviductal lesions, equivalent to serous tubal intraepithelial carcinomas in women, are usually first observed 6–8 months after tumor induction, which is around the time of mouse menopause [10,12]. Because ovarian endosalpingiosis-like lesions are known to occur in mice [13,14,15], *BRPN* mice provide a unique model to test whether HGSC can arise from non-neoplastic tubal-type epithelium outside of the mouse oviduct, and to compare the protective effects of removing the oviducts alone versus the oviducts and ovaries from mice following tumor induction. We report our studies to determine if mice whose oviducts are surgically removed shortly before tumor induction with TAM acquire HGSCs outside of the oviduct, for example, in association with ovarian endosalpingiosis-like lesions. We also report whether removal of both ovaries and oviducts from the mice after tumor induction conferred additional protection from development of HGSC beyond removing the oviducts alone. These latter studies model the effects of risk-reducing salpingectomy (RRS) versus risk-reducing salpingo-oophorectomy (RRSO) in women with known genetic predisposition to HGSC, such as carriers of *BRCA1/2* pathogenic variants.

## 2. Materials and Methods

### 2.1. Genetically Modified Mice and Animal Care

*Ovgp1-iCreER^T2^* mice and those also carrying combined floxed *Brca1*, *Trp53*, *Rb1*, *Nf1* TSG alleles (*BPRN* mice) and tumor induction with TAM have been described previously in detail [9,11,16]. Genotypes of the mice included in this study were confirmed by PCR analysis of tail-derived DNA using PCR primers reported previously [7]. The same primers were used to detect recombination of floxed TSG alleles in mouse tumors that developed in TAM-treated *BPRN* mice. Genomic DNA was isolated from formalin-fixed paraffin-embedded tumor tissues using the FFPE Tissue Kit (Qiagen, Germantown, MD, USA), following the manufacturer′s instructions, and PCR amplified using GoTaq^®^ Master Mixes (Promega, Madison, WI, USA). All procedures involving mice described in this study were approved by the University of Michigan’s Institutional Animal Care and Use Committee (PRO00008343 and PRO00010212).

### 2.2. Bilateral Salpingectomy and Salpingo-Oophorectomy

Survival surgeries were performed on mice anesthetized by isoflurane, as previously described [9,11,16]. To determine effects of bilateral salpingectomy (BS) on the frequency of developing ovarian or primary peritoneal HGSC, removal of both oviducts was performed on a cohort (*n* = 25) of 8 week old *BPRN* mice. The uterus and ovaries were left intact. Another cohort of *BPRN* mice (*n* = 27) underwent removal of oviducts and ovaries (bilateral salpingo-oophorectomy, BSO) at 8 weeks of age. In this cohort, the uterus was left intact. A smaller cohort (*n* = 7) of age-matched *BPRN* mice underwent sham surgery in which the peritoneal cavity was opened, the ovaries and oviducts were exteriorized and then returned, and surgical sites were closed. After two weeks to allow recovery from the surgical procedure, mice were transiently treated with TAM (0.2 g/kg, intraperitoneally on day 1 and day 3) to inactivate the conditional TSG alleles in oviductal-type (OVGP1-expressing) epithelium present in the ovaries, peritoneum, or other sites. Mice were followed for 70 weeks post-TAM or euthanized earlier if humane endpoints were reached. Mice were necropsied and grossly evaluated for tumor presence and extent. In each case, the ovaries and oviducts (if present), uteri, lungs, multiple abdominal organs, and omentum/abdominal fat were examined microscopically. The presence or absence of other neoplasms arising spontaneously in the aged animals was also noted.

### 2.3. Histopathology and Immunohistochemistry

Formalin-fixed and paraffin-embedded (FFPE) tissue sections were stained with hematoxylin and eosin (H&E) for evaluation by light microscopy. To identify microscopic lesions in grossly normal oviducts and ovaries, each oviduct and/or ovary was serially sectioned (5 µm intervals) in its entirety and alternate sections were either stained with H&E or retained for immunohistochemical (IHC) staining according to standard methods as described previously [9,11,16]. At least 12 H&E-stained sections representing serial levels of omentum were examined per mouse, while other organs were microscopically assessed by representative sections only. The following primary antibodies were used: rabbit anti-OVGP1 polyclonal antibody (Abcam, Cambridge, UK), rabbit anti-PAX8 (Proteintech, Chicago, IL, USA), rabbit anti-WT1 antibody (Santa Cruz, Dallas, TX, Thermo Fisher, Waltham, MA, USA), rabbit anti-acetyl tubulin (Cell Signaling, Danvers, MA, USA) and rat anti-cytokeratin 8 (CK8) (Developmental Studies Hybridoma Bank, Iowa City, IA, USA), rat anti-Ki-67 (Invitrogen, Thermo Fisher). Immunostaining of mouse tissues was scored based on the percentage and intensity of positive cells: “−”, absent in all cells; “+/−”, <10% positive cells with barely perceptible staining; “+”, 10–25% positive cells with weak staining; “++”, 26–50% positive cells with moderate staining; “+++”, >50% positive cells with strong staining.

### 2.4. Statistical Analysis

The Mantel-Haenszel Chi-square test was performed using SPSS 29 software to compare the effects of the two surgical interventions (BS or BSO) vs. sham surgery. Differences with *p* < 0.05 were considered statistically significant. Survival rates of mice in the three cohorts (sham, BS, and BSO) were estimated by Kaplan–Meier analysis using GraphPad Prism software version 10.

## 3. Results

### 3.1. Endosalpingiosis Is Present in a Subset of Mice Following Bilateral Salpingectomy

**Figure 1:**
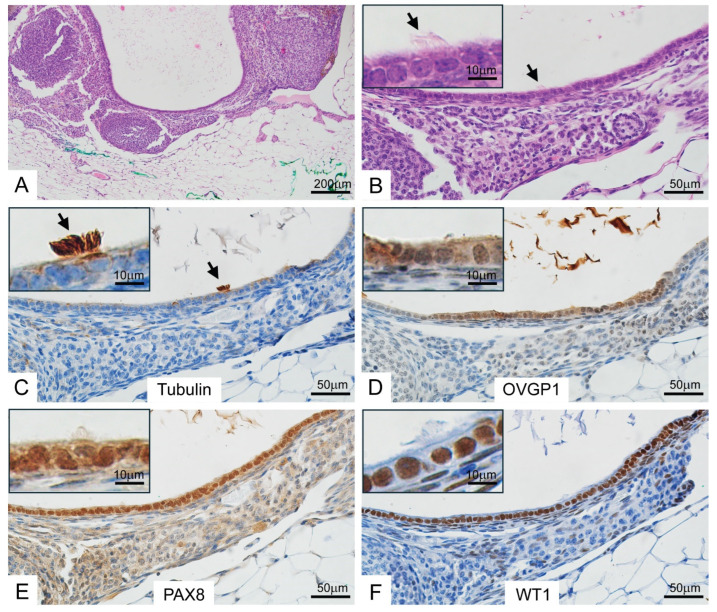
Murine ovarian endosalpingiosis expresses markers of tubal-type differentiation. H&E-stained sections of an ovary with cystic endosalpingiosis in a *BPRN* mouse treated with bilateral salpingectomy prior to TAM-induced gene targeting in oviductal epithelium (**A**,**B**). Immunohistochemical staining of sections showing expression of tubulin (**C**), OVGP1 (**D**), PAX8 (**E**) and WT1 (**F**). Black arrows indicate ciliated cells. Insets in selected panels show higher magnifications. Scale bars represent 200 µm, 50 µm and 10 µm as indicated on each panel.

In prior studies, we used *OVGP1-iCreER^T2^* mice also expressing a Cre-inducible eYFP reporter allele (*R26R^LSL-eYFP^*) to detect oviductal epithelium located outside of the oviduct, and we identified ovarian endosalpingiosis-like lesions in 14% of TAM-treated adult *OVGP1-iCreER^T2^*;*R26R^LSL-eYFP^* mice [17]. The endosalpingiotic inclusion glands and cysts were lined by secretory and ciliated cells and expressed PAX8, tubulin and OVGP1. We have also incidentally observed ovarian endosalpingiosis in the course of other work using our HGSC GEMMs (unpublished data). In this study, we identified endosalpingiosis in 6 of 50 (12%) ovaries from the 25 TAM-treated *BPRN* mice that underwent BS. A representative example is shown in Figure 1, in which a group of glands, some cystically dilated, are present in the ovarian stroma and are lined by cytologically bland epithelium (Figure 1A,B) that expresses markers of oviductal differentiation, including tubulin, OVGP1, PAX8 and WT1 (Figure 1C–F). Detailed information on the histopathologic features of endosalpingiosis and quantitative data of immunohistochemical staining are provided in Supplemental Table S1.

**Figure 2:**
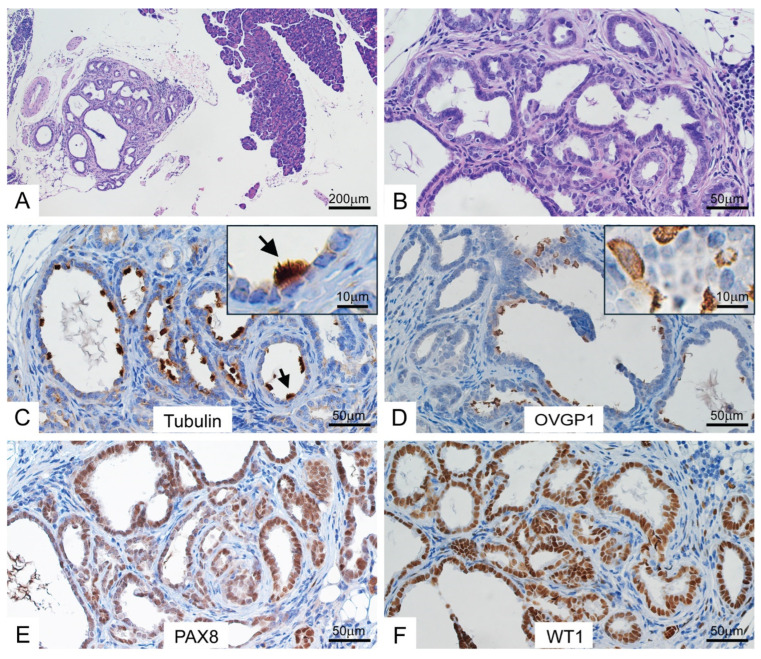
Murine peritoneal endosalpingiosis expresses markers of tubal-type differentiation. H&E-stained sections of peritoneal endosalpingiosis in a *BPRN* mouse that underwent bilateral salpingectomy prior to TAM-induced gene targeting in oviductal epithelium (**A**,**B**). Immunohistochemical staining of sections showing expression of tubulin (**C**), OVGP1 (**D**), PAX8 (**E**) and WT1 (**F**). Black arrows indicate ciliated cells. Insets in selected panels show tubulin and OVGP1 at higher magnification. Scale bars represent 200 µm, 50 µm and 10 µm as indicated on each panel.

In addition, we identified focal endosalpingiosis with similar morphological and immunohistochemical features in the omentum of one *BPRN* mouse after BS (Figure 2A–F).

In mice, endosalpingiosis involving non-ovarian tissues is likely less common than ovarian endosalpingiosis, perhaps because the mouse ovarian bursa limits movement or dispersion of detached tubal epithelium to non-ovarian tissues. Also, the incidence of non-ovarian endosalpingiosis may be underestimated due to our inability to examine the omentum, abdomino-pelvic lymph nodes, and peritoneal surfaces for these microscopic lesions in an exhaustive fashion.

### 3.2. Bilateral Salpingectomy Significantly Reduces the Incidence of Non-Tubal HGSC Development in TAM-Treated BPRN Mice, but Not as Completely as Bilateral Salpingo-Oophorectomy

**Figure 3:**
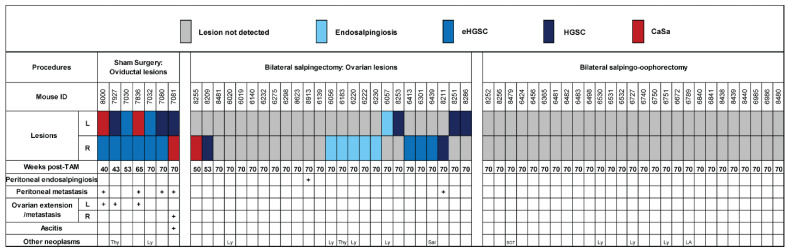
Summary of findings in TAM-treated *BPRN* mice following sham surgery, bilateral salpingectomy, or bilateral salpingo-oophorectomy. Weeks post-TAM indicates the time point at which mice were sacrificed. L, left; R, right oviduct (left section) or ovary (center section). Colored boxes indicate type of lesion identified (eHGSC: early HGSC confined to oviduct or ovary; HGSC; CaSa: carcinosarcoma). Additional tumors observed included Thy: Thyroid tumor; Ly: Lymphoma; Sar: Sarcoma; SGT: salivary gland tumor; LA: Lung adenocarcinoma.

Tumor development in cohorts of TAM-treated *BPRN* mice after undergoing sham surgery, BS, or BSO are summarized in Figure 3.

In agreement with our prior work, all *BPRN* mice that underwent sham surgery developed HGSC confined to the oviduct (eHGSC) or oviductal HGSC or carcinosarcoma (CaSa, a HGSC variant that has undergone epithelial-to-mesenchymal transition) extending beyond the oviduct, with more than half of the mice reaching humane endpoints before the end of the surveillance period (Figure 3, left section). Most of these mice had metastatic disease by study endpoint. In the cohort of 25 TAM-treated *BPRN* mice that underwent BS, 9 mice developed ovarian primary tumors over the 70-week surveillance period (Figure 3, center section). Three tumors were early HGSC (eHGSC, confined to the ovary) and six were more advanced tumors (HGSC or CaSa) that had spread beyond the ovary directly into adjacent soft tissue and/or metastasized to peritoneal surfaces. Representative gross photographs of advanced tumors arising in the ovaries of mice after BS are shown in Supplemental Figure S1. Two of the mice that developed advanced tumors reached humane endpoints earlier than 70 weeks post-TAM. None of the mice that underwent BS developed tumors with features of primary peritoneal HGSC, i.e., peritoneal disease in the absence of an ovarian tumor, although we cannot exclude the possibility of an occult peritoneal tumor given our inability to exhaustively examine the peritoneal surfaces in the mice.

**Figure 4:**
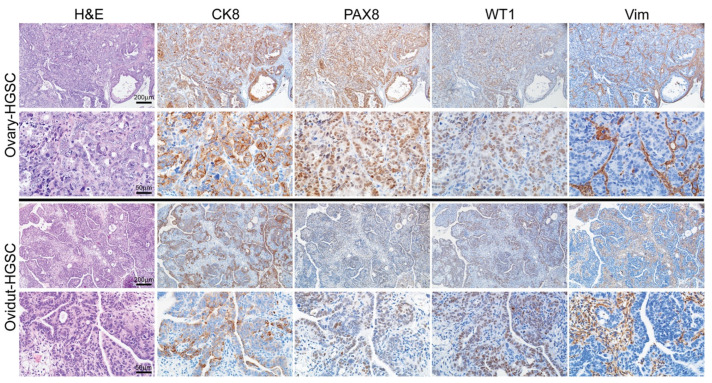
Tumors arising in the *BRPN* mice after sham surgery or bilateral salpingectomy, have comparable morphology and immunophenotype. The upper panels show H&E and immunohistochemical stains of a representative HGSC arising in the ovary of a TAM-treated *BPRN* mouse after bilateral salpingectomy. Lower panels show H&E and immunohistochemical stains of a representative HGSC arising in the oviduct of a TAM-treated *BPRN* mouse after sham surgery. Sections were immunostained using antibodies for detection of cytokeratin 8 (CK8), PAX8, WT1 and vimentin (Vim). Scale bars represent 200 µm and 50 µm as indicated.

As expected, tumors that developed in the ovaries after BS (Figure 4 upper panels) are morphologically comparable to the oviductal tumors that arose in the sham surgery cohort (Figure 4, lower panels), express markers characteristic of HGSCs—including CK8, PAX8, and WT1, and display recombination of the targeted TSGs (Supplemental Figure S2). In contrast, no peritoneal or other tumors were observed in any of the 27 mice that underwent removal of both oviducts and ovaries (Figure 3, right section). Statistical analysis showed that compared with the sham surgery group, tumor development was significantly reduced by both BS and BSO (*p* < 0.001 for both groups by the Mantel-Haenszel Chi-square test), although only the latter afforded complete protection from tumor development. Kaplan–Meier survival analysis of the mice in this study is shown in Supplemental Figure S3. Our findings on the effects of BS versus BSO in our *BRPN* GEMM provide unique insights regarding the limited protection against cancer development afforded by RRS.

### 3.3. HGSC Is Associated with Endosalpingiosis in Mice

**Figure 5:**
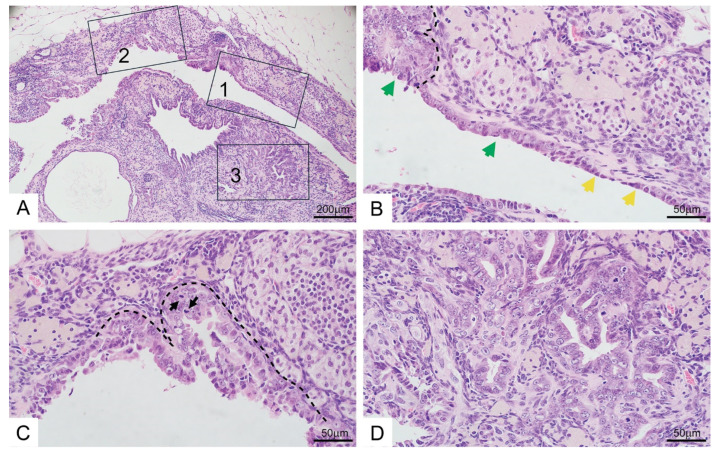
HGSC is associated with and appears to arise from ovarian endosalpingiosis in a TAM-treated *BPRN* mouse after bilateral salpingectomy. (**A**) Low-magnification photomicrograph of H&E-stained section of ovary showing eHGSC in continuity with endosalpingiosis. Boxed areas 1–3 are shown at higher magnification in (**B**–**D**) panels, respectively. (**B**) Area in Box 1 showing transition of benign-appearing oviductal-type epithelium (yellow arrows) to highly atypical epithelium (green arrows) consistent with serous intraepithelial carcinoma (SIC). (**C**) Area in Box 2 showing SIC lesion. Black arrows indicate mitotic figures. (**D**) Area in Box 3 showing invasive HGSC, still confined to ovary (eHGSC). Scale bars represent 200 µm and 50 µm as indicated.

As noted above, some HGSCs in women likely arise outside of the fallopian tubes. The cells of origin for non-tubal HGSC lesions remain unclear. In our studies, an eHGSC arising in the ovary of a salpingectomized mouse appeared to arise from associated cystic endosalpingiosis. Figure 5 shows representative photomicrographs of the ovary in the mouse with an eHGSC lesion.

The ovary shows endosalpingiosis in which benign-appearing epithelium is in continuity with epithelium displaying marked cytologic atypia (in situ carcinoma, referred to here as serous intraepithelial carcinoma, SIC), and then transitions to locally invasive carcinoma (eHGSC). Some markers of tubal differentiation present in the endosalpingiosis such as tubulin and OVGP1 are diminished in the invasive carcinoma, which retains expression of WT1, PAX8. Increased Ki-67 positive cells were observed in the SIC and eHGSC lesions (Supplemental Figure S4). Our observation of HGSC arising from endosalpingiosis provides a plausible explanation for why even salpingo-oophorectomy does not afford complete protection in women at high-risk of HGSC development [18].

## 4. Discussion

Most ovarian cancer-associated deaths among women in the United States are attributable to HGSC and there is general consensus that most HGSCs arise in the fallopian tubes—specifically from precursor lesions known as serous tubal intraepithelial carcinomas (STICs). Based on comprehensive DNA, RNA, and miRNA profiling, Ducie and colleagues showed that the molecular features of HGSCs with and without associated STIC lesions are mostly shared, supporting a common biologic origin they favored to be the distal fallopian tube [19]. Notably, however, when correlating RNA expression data between HGSCs and normal tissues, they found nearly 12% of HGSCs were better correlated to normal ovarian surface epithelium (OSE) than to normal FTE, supporting potential ovarian origin. Using transgenic mice, Zhang and colleagues showed that HGSCs can arise from either FTE or OSE and comparison of FTE- and OSE-derived mouse tumors to human HGSCs suggested that HGSCs can arise from both cell types [2]. Because the subset of HGSCs apparently arising outside of the fallopian tube (e.g., ovary or peritoneum) usually present at advanced stage [20], only a few STIC-like lesions or early HGSCs arising outside of the tubes have been reported, for example, in ovarian serous cystadenoma, serous cystadenofibroma, or benign serous cysts [3,4,21]. Collectively, these data support the conclusion that while most HGSCs indeed arise from the fallopian tube, a subset does not. However, it is still unclear from which cells these non-tubal HGSCs arise. Dubeau has suggested the “secondary Müllerian” system including endometriosis, endosalpingiosis, and paraovarian/paratubal cysts, may be playing roles in the pathogenesis of Mullerian carcinomas that appear to arise outside of the primary Mullerian tract [22,23]. Primary peritoneal HGSCs have been reported in women decades after BSO. Because the time needed for progression from STIC to HGSC is much shorter (approximately 6 years) [24], these tumors are more likely arising from endosalpingiosis [25,26,27]. A recent systematic review of peritoneal HGSCs arising after risk-reducing surgery concluded that most available evidence supports the dissemination of precursor cells originating in the fallopian tube, with ovarian origin considered less likely [28]. The dissemination of precursor cells could include not only benign tubal epithelium (i.e., endosalpingiosis), but also neoplastic epithelium from STICs and other early serous proliferations in the tube including p53 signature lesions through “precursor escape” [5,29].

HGSC GEMMs, in which the timing of tumor development is known and under experimental control, provide an ideal system with which to test if HGSCs can arise outside of the oviduct, and from what types of precursors. We found that removal of both oviducts prior to tumor induction markedly reduced but did not completely eliminate the development of HGSC, and the tumors are likely arising from ovarian endosalpingiosis lesions already present at the time of transient treatment with TAM to induce tumor formation. In contrast, removal of both oviducts and ovaries was completely protective, perhaps because endosalpingiosis in mice is predominantly found in the ovaries, which were removed prior to TAM treatment. We also observed a case of ovarian endsalpingiosis contiguous with SIC and invasive HGSC, providing histopathologic evidence for endosalpingiosis as a precursor from which non-tubal HGSCs can arise. While our findings provide support for the superiority of BSO over BS alone in preventing the development of HGSC, a limitation is that findings based solely on the *BPRN* model system may not be generalizable to other models not tested here. Moreover, anatomical differences between mice and humans may have impacted our findings compared to those expected in humans. Because dissemination of tubal epithelium (normal or neoplastic) to the peritoneal cavity but not the ovary is prevented by the ovarian bursa, it is not surprising that we have never identified presumptive primary peritoneal HGSC in *BPRN* mice. While BS and BSO are expected to disrupt the bursa, the timing of surgical intervention and TAM treatment could also impact the frequency and distribution of sites at which tumors arise.

In the general population, opportunistic salpingectomy is associated with reduced ovarian cancer risk [30]. For women with known genetic predisposition for developing HGSC (e.g., *BRCA1/2* mutation carriers), primary prevention strategies include RRSO, RRS, and salpingectomy with delayed oophorectomy (SDO) [31,32,33]. In high-risk populations RRSO has been shown to be very effective in reducing the risk of ovarian cancer [27,34], and in a recent study of more than 2500 women with germline pathogenic variant *BRCA1/2* alleles who underwent RRSO, the 20-year cumulative incidence rate of HGSC was 1.5% for *BRCA1* and 0.2% for *BRCA2* [18]. RRS, while less studied, is being investigated as a potential interim measure for younger at-risk women who wish to delay the onset of menopause but still want to reduce cancer risk. Our findings support the conclusion that RRSO affords more protection from developing HGSC than RRS alone. Importantly, our work does not address whether RRS with delayed oophorectomy might offer substantial HGSC risk-reduction while avoiding the consequences of inducing premature menopause. Experiments could be done in which cohorts of mice undergo BS, BSO or SDO at defined intervals after TAM exposure to determine whether and how the type and timing of surgery might influence protective effects and/or tumor distribution, and the results could inform studies in humans. Several ongoing clinical trials (NCT04294927, NCT04251052, and ISRCTN25173360) are evaluating the long-term outcomes and effectiveness of RRS or SDO versus RRSO, particularly in relation to quality of life, cancer risk reduction, and overall survival [35,36,37].

## 5. Conclusions

The current study illustrates the impact of GEMMs in advancing a more complete understanding of HGSC pathogenesis and how this knowledge can potentially be used to optimize prevention strategies for both the at-risk and general populations. Our findings provide experimental evidence for the superiority of RRSO relative to other strategies for reducing the risk of HGSC, while also providing a basis for further investigation of alternative preventive measures, including salpingectomy with delayed oophorectomy.

## Data Availability

Data is contained within the article or Supplementary Material—The original contributions presented in this study are included in the article/Supplementary Material. Further inquiries can be directed to the corresponding author.

## Supplementary Materials

The following supporting information can be downloaded at: https://www.mdpi.com/article/10.3390/cancers17172759/s1, Table S1: Summary of Endosalpingiosis, eHGSC and HGSC/CaSa Histopathology and Immunohistochemical Staining; Figure S1: Gross photographs of representative ovarian (Ov) tumors arising in TAM-treated *BPRN* mice after bilateral salpingectomy; Figure S2: Genotyping of mouse HGSCs arising in the ovaries of *BPRN* mice after bilateral salpingectomy; Figure S3: Kaplan-Meier survival analysis of mouse cohorts following sham surgery (red: *n* = 7), BS (green: *n* = 25), or BSO (blue; *n* = 27); Figure S4: Representative photomicrographs of endosalpingiosis and contiguous eHGSC in the ovary of mouse #6413 immunostained for markers of tubal-type differentiation.

## Abbreviations

The following abbreviations are used in this manuscript:

HGSC	High-grade serous carcinoma
GEMM	Genetically engineered mouse model
*BPRN*	Brca1, Trp53, Rb1, and Nf1
TSGs	Tumor suppressor genes
FTE	Fallopian tube epithelium
RRS	Risk-reducing salpingectomy
RRSO	Risk-reducing salpingo-oophorectomy
STICs	Serous tubal intraepithelial carcinomas
eHGSC	Early High-grade serous carcinoma
SIC	Serous intraepithelial carcinoma
CaSa	Carcinosarcoma
SDO	Salpingectomy with delayed oophorectomy
