# Supplementary material for: Bilateral Salpingo-Oophorectomy Is Superior to Salpingectomy Alone in Preventing Non-Tubal Tumor Development in a Mouse Model of High-Grade Serous Carcinoma
Source: Cancers (Basel). 2025 Aug 24;17(17):2759. doi: 10.3390/cancers17172759 (PMC12427576; doi:10.3390/cancers17172759)
Supplement: Supplementary file 1 [file cancers-17-02759-s001.zip › Supplemental Table-1.pdf]

Supplemental Table-1. Summary of Endosalpingiosis, eHGSC and HGSC/CaSa Histopathology and Immunohistochemical Staining

ID	Surgery	Weeks Post TAM	Histopathology		IHC					
			Left ovary	Right ovary	Tubulin	Ovgp1	Pax8	WT1	CK8	VIM
6056	*BPRN*/RRS	(70W)	no lesion	endosalpingiosis	+	+	+	++	NA	NA
6230	*BPRN*/RRS	(70W)	no lesion	endosalpingiosis	+	+	±	_	NA	NA
6057	*BPRN*/RRS	(70W)	no lesion	endosalpingiosis	_	+	+	++	NA	NA
6222	*BPRN*/RRS	(70W)	no lesion	cystic endosalpingiosis	±	±	+	++	NA	NA
6220	*BPRN*/RRS	(70W)	no lesion	cystic endosalpingiosis	+	+	++	+++	NA	NA
6183	*BPRN*/RRS	(70W)	atypical endosalpingiosis, papillary spectrum	no lesion	+	+	+	++	NA	NA
8913	*BPRN*/RRS	(70W)	peritoneal endosalpingiosis		++	+	++	+++	NA	NA
6413	*BPRN*/RRS	(70W)	no lesion	cystic endosalpingiosis & eHGSC shows full spectrum	+	+	+++	++	+	NA
6439	*BPRN*/RRS	(70W)	no lesion	eHGSC	+	+	+	+	++	NA
6301	*BPRN*/RRS	(70W)	no lesion	eHGSC &cystic portion possible representing transformation in ES	_	±	+	+	++	NA
8209	*BPRN*/RRS	(53W)	no lesion	HGSC, psudoendometrioid	NA	NA	++	++	++	+
8211	*BPRN*/RRS	(70W)	no lesion	HGSC	NA	NA	+++	++	++	++
8286	*BPRN*/RRS	(70W)	solid and papillary HGSC adjacent ES	no lesion	NA	NA	++	+++	+	++
8251	*BPRN*/RRS	(70W)	no lesion	HGSC, full spectrum	NA	NA	++	++	++	+
8253	*BPRN*/RRS	(70W)	Cystic HGSC	no lesion	NA	NA	++	++	+	+
8255	*BPRN*/RRS	(50W)	no lesion	CaSa	NA	NA	±	±	±	+++
