# Supplementary figures and images for: Bilateral Salpingo-Oophorectomy Is Superior to Salpingectomy Alone in Preventing Non-Tubal Tumor Development in a Mouse Model of High-Grade Serous Carcinoma
Source: Cancers (Basel). 2025 Aug 24;17(17):2759. doi: 10.3390/cancers17172759 (PMC12427576; doi:10.3390/cancers17172759)

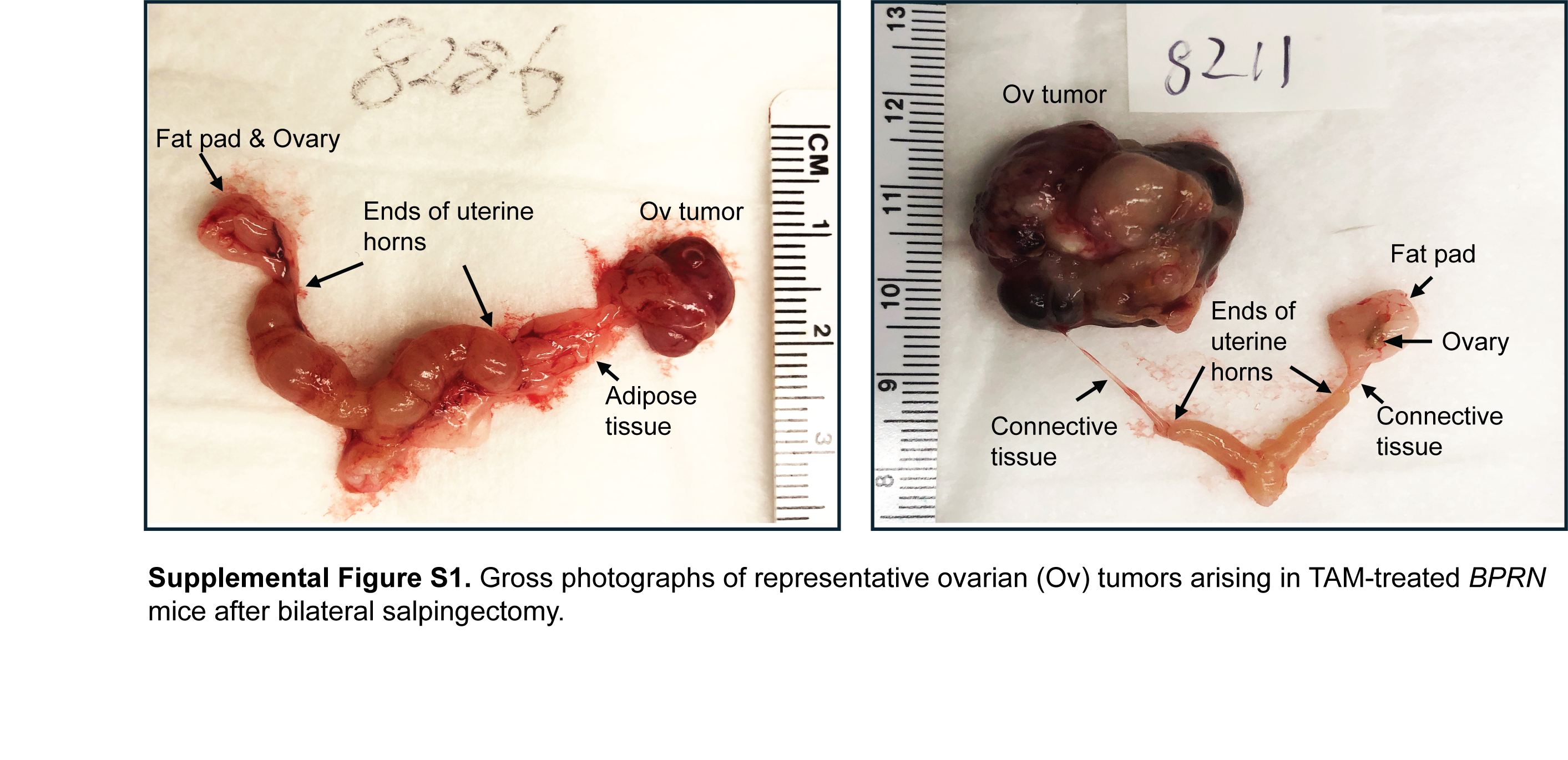

Supplement: Supplementary file 1 [file cancers-17-02759-s001.zip › S-Figure-1.tif]

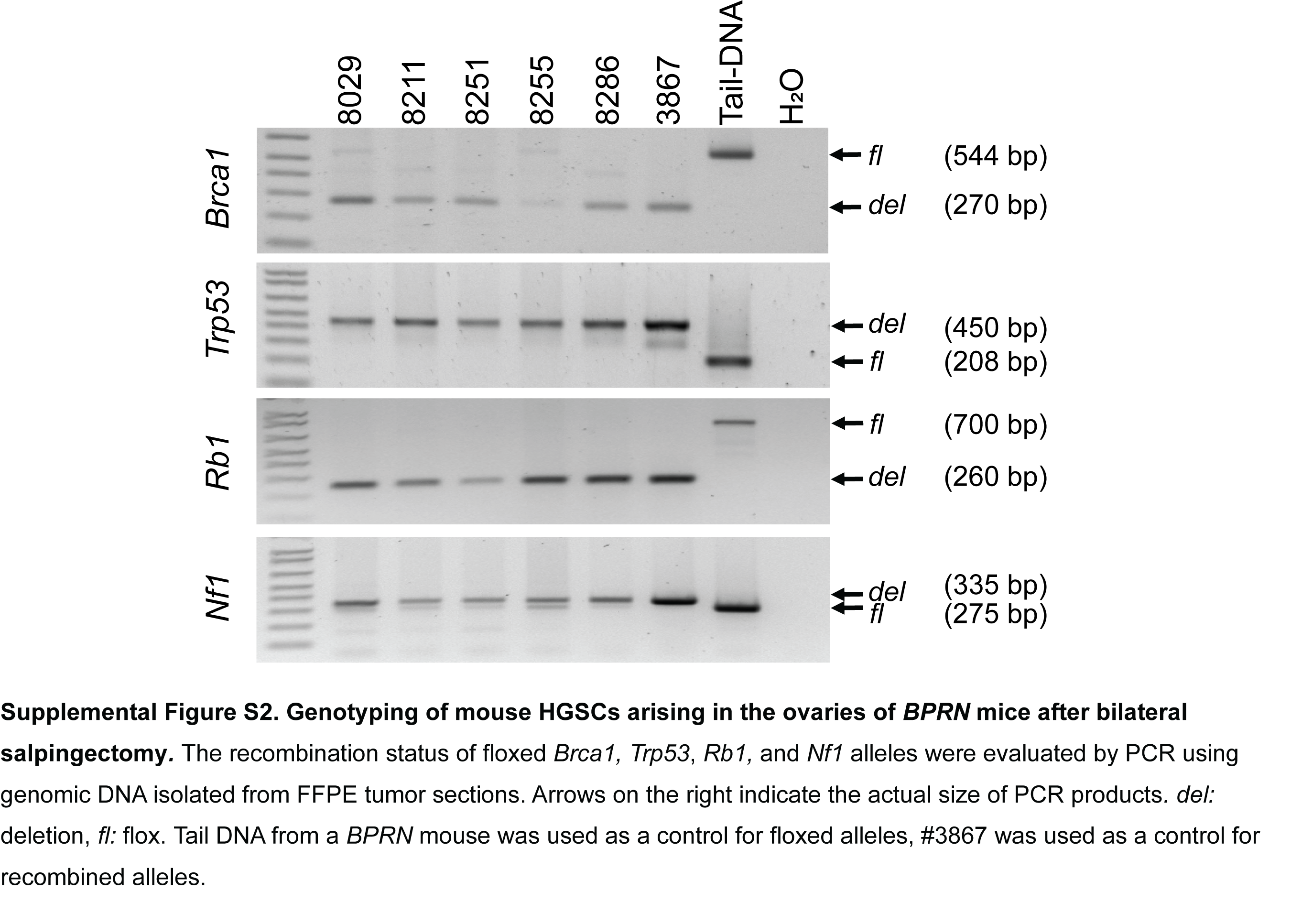

Supplement: Supplementary file 1 [file cancers-17-02759-s001.zip › S-Figure-2.tif]

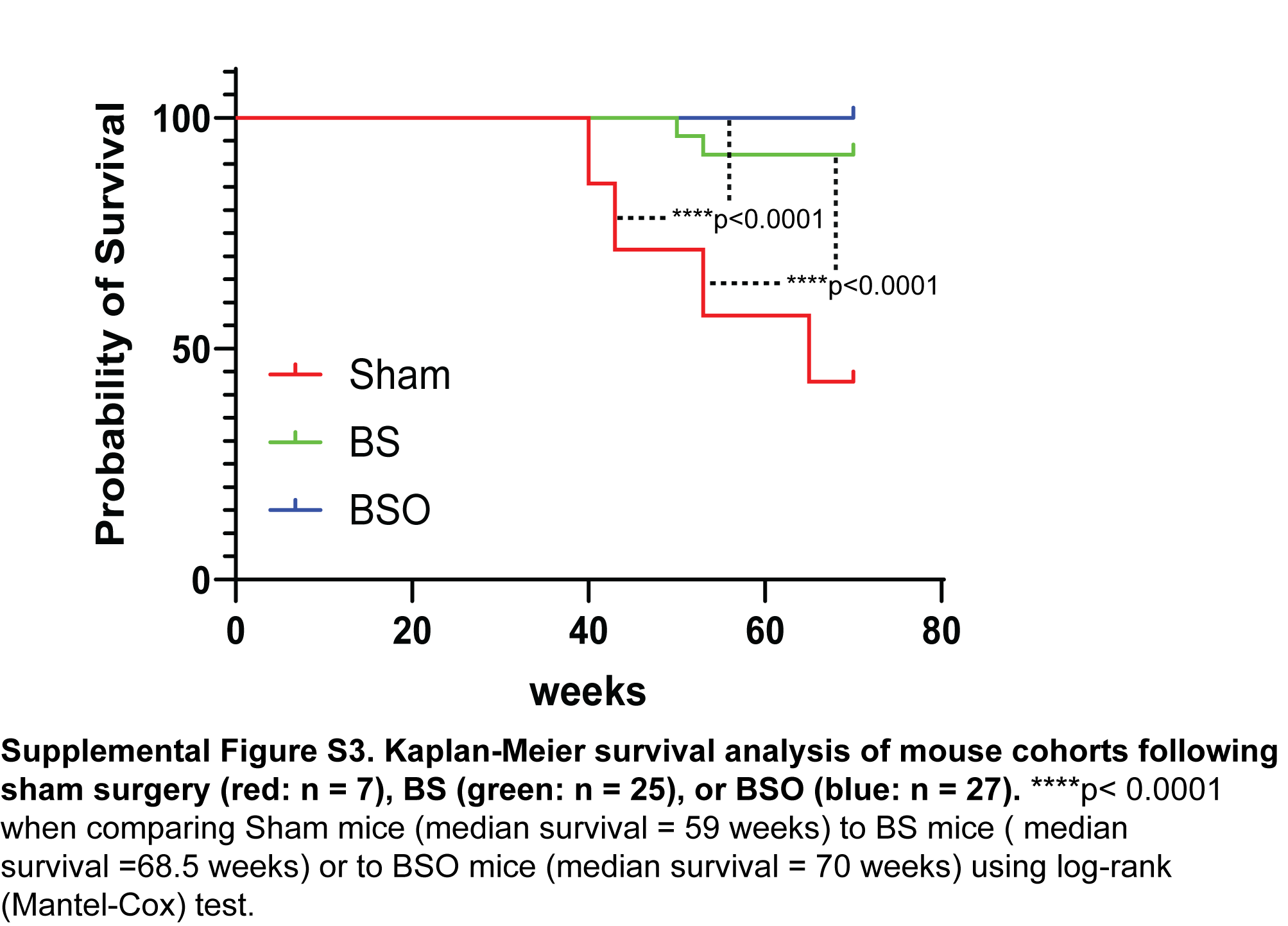

Supplement: Supplementary file 1 [file cancers-17-02759-s001.zip › S-Figure-3.tif]

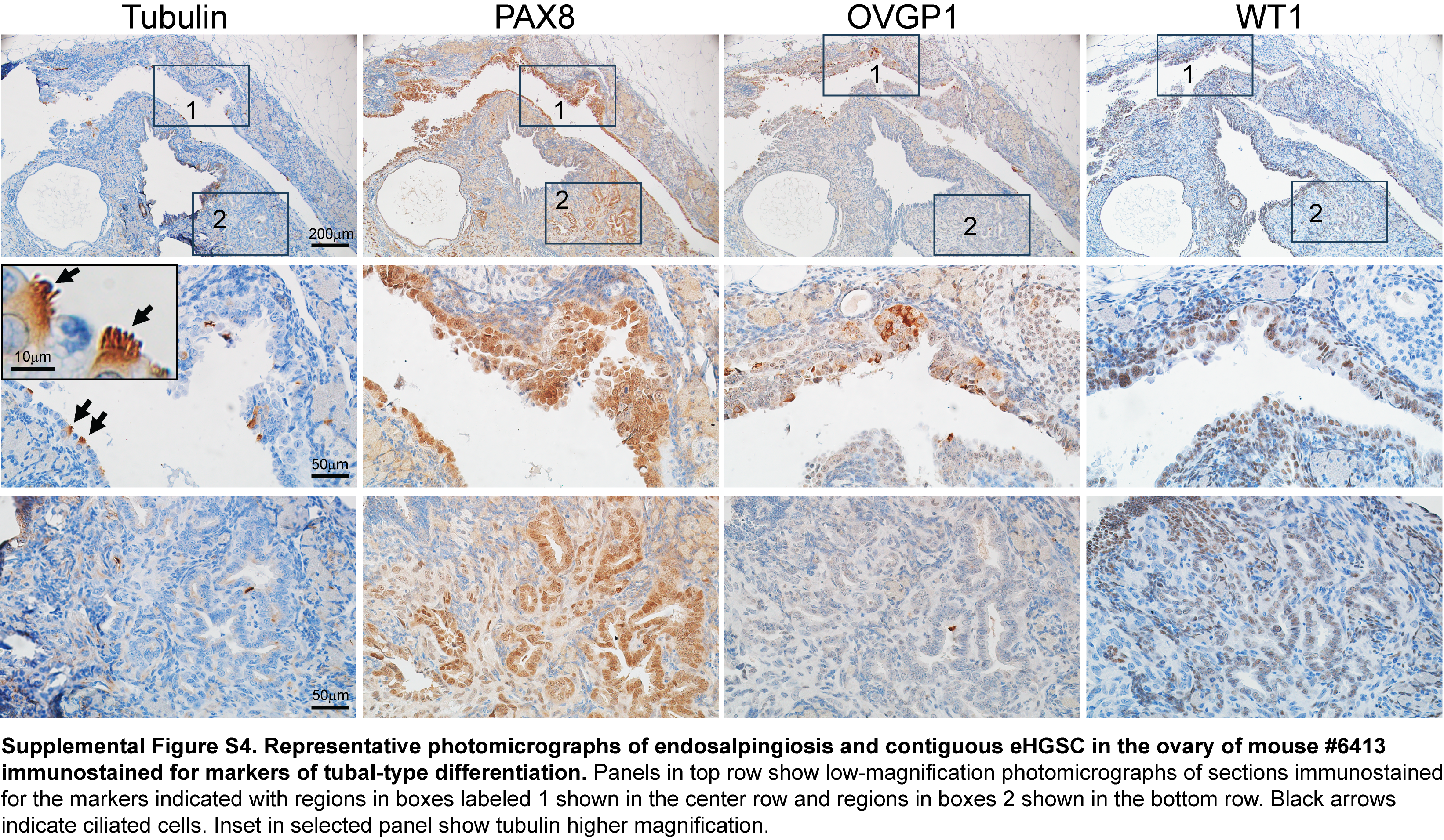

Supplement: Supplementary file 1 [file cancers-17-02759-s001.zip › S-Figure-4.tif]
